# High density lipoprotein: When to rethink too much of a good thing

**DOI:** 10.1016/j.ajpc.2023.100511

**Published:** 2023-06-22

**Authors:** Lily N. Dastmalchi, Charles A. German, Pam R. Taub

**Affiliations:** a Section of Cardiology, Department of Medicine, Temple University Hospital, Philadelphia, PA, USA; b Section of Cardiology, Department of Medicine, University of Chicago, Chicago, IL, USA; c Section of Cardiology, Department of Medicine, University of California San Diego, San Diego, CA, USA

**Keywords:** High density lipoprotein, Cholesterol efflux capacity, Gestational hypertension, Menopause

## Abstract

High density lipoprotein cholesterol (HDL-C) is a known contributor to atherosclerotic cardiovascular disease (ASCVD) risk when HDL-C <40 mg/dL in men and <50 mg/dL in women. There has been much interest in the potential cardioprotective properties of HDL-C, as it removes cholesterol from the periphery to the liver for exertion and holds inherent anti-thrombotic and anti-inflammatory properties. However, clinical trials raising HDL-C pharmacologically have not shown to improve cardiovascular outcomes. In fact, observational studies have demonstrated an increased risk of non-cardiovascular mortality and infection when HDL-C >90 mg/dL and >70 mg/dL in women and men, respectively. The ability for the HDL particle to effectively transport cholesterol from the periphery for excretion in bile is more complex than illustrated on a standard cholesterol panel. There is variability in its function, size, density, subclass, reverse cholesterol transport, and cholesterol efflux capacity, which impact the particles ability to effectively reduce cardiovascular disease (CVD) risk. Research has shown that HDL particles are prone to have a reduction in its efficacy in response to infection, auto-immune disease, menopause and cardiometabolic conditions during pregnancy. Additionally, recent studies have shown that low HDL-C may not adequately influence ASCVD risk in Black adults. The purpose of this contemporary review is to highlight the utility of using HDL-C in assessing CVD risk.

## 1 Introduction

Low levels of high density lipoprotein (HDL) cholesterol (HDL-C), high lipoprotein (LDL) cholesterol (LDL-C) and high triglycerides were first associated with the development of coronary heart disease in the mid 20th century [1,2]. In the 1970s, the Framingham Heart Study observed an inverse relationship between HDL-C and cardiovascular disease (CVD) risk [2,3]. Low HDL-C is a well-defined risk factor of both incident and progression of atherosclerosis [1,[4], [5], [6]]. Alternatively, high HDL-C is thought to be cardioprotective, due to greater cholesterol uptake from the periphery and excretion in the bile. Additionally, HDL particles are thought to have anti-oxidative, anti-inflammatory, and anti-thrombotic effects, thus further inferring cardiovascular benefit [4,7]. For each 1 mg/dL increase in HDL-C, there is a measured reduction in CVD mortality by 4.7% in women and 3.7% in men [8]. Given the strong association between HDL-C and CVD risk, HDL-C is a core component of the pooled cohorts equation, the gold standard in assessing 10-year atherosclerotic cardiovascular disease (ASCVD) risk in the United States. Using this equation, a higher HDL-C reduces ASCVD risk, though more contemporary data has shed light on the potential negative effects of high HDL-C. Observational studies highlighting an inverse association between very high HDL-C and cardiovascular outcomes, coupled with randomized controlled trials demonstrating little cardioprotective benefit when pharmacologically increasing HDL-C, have called into question the benefit of high HDL-C [9]. The purpose of this review is to summarize the most up to date literature regarding the relationship between high HDL-C and ASCVD, while also proposing an algorithm to guide clinical management.

## 2 Pharmacologically raising HDL-C

Randomized controlled trials have demonstrated that increasing HDL-C pharmacologically does not improve cardiovascular outcomes [5,10]. High dose niacin has several differential effects on lipoproteins levels: it increases HDL-C, decreases plasma triglycerides, lipoprotein (a), and impairs hepatic synthesis of very low density lipoprotein [11]. Additionally, niacin decreases LDL-C levels by at least 19% [11]. Given these beneficial properties, the Atherothrombosis Intervention in Metabolic Syndrome with Low HDL/High Triglycerides: Impact on Global Health Outcomes (AIM-HIGH) trial was conducted to understand the utility of niacin among patients with low HDL-C, which enrolled over 3000 patients on high intensity statin therapy. Despite favorable effects on HDL-C and triglyceride levels, niacin did not result in a decrease in major adverse cardiovascular events [12]. Similarly, the Heart Protection Study-2 Treatment of HDL to Reduce the Incidence of Vascular Events (HPS2-THRIVE) also found that high dose niacin with laropiprant (which reduces niacin associated flushing and improves adherence) did not reduce major adverse cardiovascular events compared with placebo [13].

Cholesterol ester transfer proteins (CETP) enable the transfer of cholesterol ester from HDL to apolipoprotein B (apoB) associated lipoproteins, leading to a decrease in HDL-C and an increase in LDL-C levels. Observational studies have demonstrated that individuals with a loss-of-function mutation in CETP had a reduction in CVD events via reduction in LDL-C and apoB levels [11,14]. The first of the pharmacologic CETP inhibitors developed, torcetrapib, was found to increase HDL-C levels by 70% and reduce LDL-C levels by 25% among patients on background statin therapy. However, there was no reduction in CVD events, with subsequent termination of the trial as the drug impacted aldosterone levels causing a rise in blood pressure [9,11,14]. Another CETP inhibitor, dalcetrapib, increased HDL-C levels without significantly effecting LDL-C levels [14]. In a study enrolling high risk patients within 12 weeks of an acute coronary syndrome, dalcetrapib increased HDL-C levels by roughly 25%, though a reduction in CVD events did not occur [14]. Lastly, anacetrapib reduced cardiovascular events and lowered CVD risk in clinical trials. However, subsequent studies found that it's mechanism for CVD reduction was primary driven by the reduction in LDL-C and apoB, rather than an increase in HDL-C [9,11,15]. Evacetrapib reduces LDL-C levels and increases HDL-C levels with a neutral impact on blood pressure, but the trial that tested its efficacy was stopped at 26 months due to lack of reduction in mortality [11]. In Phase II testing of obicetrapib, the Randomized Study of Obicetrapib as an Adjunct to Statin Therapy (ROSE) found up to a 30% reduction in apoB and a 51% reduction in LDL-C when obicetrapib was taken with a high-intensity statin, however CVD outcomes trials are ongoing [16]

## 3 Elevated high density lipoprotein-C

Large observational studies including High-Density Lipoprotein Cholesterol and Cause-Specific Mortality in Individuals Without Previous Cardiovascular Conditions: The CANHEART Study have illustrated that significantly elevated HDL-C infers an increased risk of mortality, which challenges the dogma that elevations in HDL-C are beneficial [17]. An HDL-C >90 mg/dL in women and > 70 mg/dL in men was associated with higher rates of mortality from cardiovascular and non-cardiovascular disease, respectively [10]. Elevated HDL-C levels may also increase the susceptibility to infection and increases rates of acute macular degeneration. Individuals with very high HDL-C are thought to have dysfunctional HDL particles, which are postulated to be pro-inflammatory and pro-atherogenic [9,18].

The current ACC/AHA ASCVD risk calculator allows for HDL-C up to 100 mg/dL, while the European Society of Cardiology (ESC) ASCVD risk calculator allows for HDL-C up to 90 mg/dL [19,20]. These values, however, do not adequately account for the role gender and race play in the development of CVD when HDL-C is extremely elevated. A retrospective study showed that HDL-C>90 mg/dL among males had an increased 10 year associated risk of major adverse cardiac events, whereas women had a similar risk at HDL-C >130 mg/dL [21]. Variability in HDL-C amongst race and ethnic groups have been identified, but their implications are unknown. For example, South Asians and Hispanics have lower HDL-C levels when compared to White and Chinese adults [22]. In the Reasons for Geographic and Racial Differences in Stroke (REGARDS) longitudinal cohort study, low HDL-C was found to predict the risk of coronary heart disease in White, but not Black adults. High LDL-C and triglyceride levels, however, were more predictive of coronary heart disease risk in both races and genders [3].

## 4 High density lipoprotein biological function and structure

With conflicting data on the relationship between HDL-C and CVD risk, there is utility in understanding the complex nature of HDL particle function beyond cholesterol transport and excretion. The HDL-C reflected on the standard cholesterol panel does not fully encapsulate the complexity of HDL particle function, where its cardioprotective benefit is reflective of the relationship between its structure and function [7,9]. The density, protein content, and size contribute to the heterogeneity of HDL particles and as a result, its ability to efficiently remove cholesterol from the periphery and transport to the liver, thereby reducing serum cholesterol and theoretically lowering the risk of CVD [5,10].

The density of HDL is determined by its protein content. These particles are considered “high-density” because of a larger percentage of protein in comparison to lipid, which are its two major constituents [4,9]. The most abundant surface protein, apolipoprotein A-I (apoA-I), makes up 70% of the HDL particle and plays a pivotal role in its affinity for cell surface receptors. ApoA-1 facilitates the removal of excess cellular cholesterol through the ATP-binding cassette transporter-1 and initiates a cascade where several other proteins are activated to help facilitate cholesterol transport [1,9]. When apoA-I binds to cholesterol molecules, it changes the composition of the particle which aides in packing and transportation to the liver for cholesterol excretion [24]. Smaller proteins further subclassified as lipoprotein-specific proteins and ancillary proteins, may play roles in facilitating the HDL particles ability to suppress inflammation and increase the effectiveness of removing cholesterol from the periphery [9].

HDL particles can be further subclassified by the size of the particle by lighter, larger and lipid-rich HDL2 and heavier, smaller, protein-rich HDL3 [25]. The size of the particle is instrumental to the function as it plays a role in cholesterol transport. Larger sized HLD particles carry larger amounts of cholesterol and bind to receptors that transfer cholesterol to the liver [4]. Since smaller HDL particles are protein rich, they have increased apoA-1 which activates enzymes involved in reverse cholesterol transport [25,26]. Generally, larger HDL particles are inversely associated with CVD risk, whereas smaller HDL particles are associated with an elevated risk of CVD. Studies have also shown that individuals identified as low risk for CVD with large HDL particle size (>8.2 nm) had lower carotid intimal thickness [27,28]. However, this relationship may be modified in older women during menopause transition, where large HDL particles may increase the risk of atherosclerosis [29]. Further research needs to be done to determine how particle size influences CVD risk.

## 5 HDL and reverse cholesterol transport

HDL particles impart cardiovascular benefit by promoting reverse cholesterol transport pathways, removing cholesterol from macrophages, enabling transport in the plasma, uptake by the liver and ultimately excretion in bile [30,31]. The capacity of HDL to remove cholesterol ester from the periphery is determined by HDL-cholesterol efflux capacity (HDL-CEC), which is the first step in reverse cholesterol transport [25,31,32]. HDL-CEC is measurable and defines how much cholesterol the HLD particle can accept from macrophages [32,33]. Additionally, these pathways have anti-inflammatory and anti-atherogenic effects mediated by suppression of hematopoietic stem cells, thereby reducing inflammation and inflammasome activation [31]. HDL-CEC has also shown benefit in protection against LDL induced apoptosis and stimulation of nitric oxide synthase to promote endothelial repair and induce angiogenesis [30]. Regardless of HDL-C content, HDL-CEC has been shown to have an independent, inverse relationship with coronary and carotid atherosclerosis [25,32].

The mechanism to which HDL-CEC functions to provide cardiovascular benefit is mainly propagated by ATP Binding Cassette A1, which mediates cholesterol efflux to apoA-I and small HDL particles and ATP binding cassette G1, which allows cholesterol efflux to mature HDL [31,34]. In vivo studies have shown accelerated atherogenesis in mice with deficiencies of both transporters, supporting the role of HDL-CEC in determining ASCVD risk. Two observational studies, the Dallas Heart Study and EPIC—Norfolk study, both found that increased levels of HDL-CEC were associated with decreased risk of ASCVD events at 9.4 years and 15 year of follow up, respectively [35,36].

## 6 HDL function and systemic disease

Chronic inflammatory conditions, like coronary artery disease, diabetes mellitus and rheumatologic disorders impart oxidative modification of apoA-I, thereby impacting reverse cholesterol transport [3,33,37]. Additionally, HDL particles also consist of triglycerides and phospholipids, where the percentage of these lipids play a role in the function of HDL-CEC [4,32]. A higher triglyceride content with a lower phospholipid content has been associated with increased cardiovascular disease [32]. One study evaluated the composition of HDL in patients with recent myocardial infarction; interestingly, their HDL particles were smaller in size and predominately composed of triglyceride [38].

Anti-inflammatory properties have also been attributed to HDL [37]. However, in infectious states and other inflammatory conditions, there is a reduction in the level of antioxidant enzymes and cholesterol content which may result in a reduction of its protective properties against atherosclerosis development [33,37]. In these circumstances, inflammatory proteins can infiltrate HDL causing a decrease in apoA-I and subsequent poor cholesterol clearance [37]. In a study of lipid function among women diagnosed with systemic sclerosis, there was an inverse relationship between severity of disease and HDL-CEC function, which may partially explain why these populations are at greater risk for coronary artery disease [33]. We composed a Table 1 that highlights the impact of chronic disease states on the HDL particle.

**Table 1:** Medical conditions that can alter HDL particle function.

Condition	Affect on HDL Particle
Infection	Reduced antioxidant potential
Autoimmune Disorders	Reduced ApoA-I
Systemic Sclerosis	Reduced HDL-CEC
Menopause	Increased HDL particle size without increase in ability to hold cholesterol
	Decreased reverse cholesterol transport
Gestational Diabetes	Decreased ApoA-I activity
	Decreased HDL-CEC
Gestational Hypertensive Disorders	Increased HDL particle size
	Impaired antioxidant function
Metabolic Syndrome	Decreased HDL-C
Diabetes	Impaired endothelial protection
	Impaired antioxidant function
	Decreased ApoA-I activity
	Decreased HDL-CEC
	Impaired anti-inflammatory properties

## 7 HDL in menopause and gestational hypertensive disorders

Post-menopausal women are at greater risk for coronary artery disease, which may be mediated by dysfunction HDL. Among perimenopausal women, larger HDL particles were found to be less effective in cholesterol transport from macrophages because of reductions in HDL-CEC in each particle [32]. It is also hypothesized that larger HDL particles are packed with cholesterol ester which may inhibit them from participating in reverse cholesterol transport [39]. HDL dysfunction is not limited to post-menopausal women, as pre-menopausal women are found to have dysfunctional HDL particles postpartum. Gestational hypertensive disorders, such as preeclampsia and gestational diabetes, are well established risk factors for the development of coronary artery disease and stroke [40,41]. Women with a history of preeclampsia are two times more likely to develop coronary artery disease and four times more likely to develop heart failure than women who did not have these conditions during pregnancy [40]. One study showed that women with gestational diabetes had decreased activity of apoA-I, which could ultimately decrease the cardioprotective benefit of HDL [23]. Additionally, these populations are found to have larger HDL particle diameters, which have impaired antioxidant function and are vulnerable to oxidation in comparison to the smaller subclasses [23,32]. Finally, gestational diabetes may lead to dysfunctional HDL from oxidation and decreased HDL-CEC [23].

## 8 Advanced HDL testing

A standardized method of evaluating HDL solely on cholesterol content (HDL-C) may not accurately reflect the particles relationship with CVD risk. HDL concentration and size can be measured using ion mobility assays and nuclear magnetic resonance (NMR) spectroscopy, respectively [1,3,28,42]. HDL measurement via NMR has been shown to be more valuable in determining CVD risk in large cohorts, however this modality is not available universally and expensive [42]. Similarly, HDL protein content can now be assessed to determine the amount of apoA-I and other subspecies by various methods of liquid chromatography and mass spectroscopy. However their utility in CVD risk stratification has not been determined [43,44].

## 9 Conclusion

In summary, the cholesterol content of the HDL alone does not fully encompass the particles association with atherosclerosis and may thereby falsely underestimate ASCVD risk, particularly when HDL-C is extremely elevated. HDL is prone to modification in response to inflammatory and immune modulating conditions such as infection, diabetes mellitus, gestational hypertensive disorders, rheumatological disorders and menopause. Advanced lipid testing assessing HDL particle number, size, and subclasses may prove beneficial in the future, given the heterogeneity of the lipoprotein particle and its impact on reducing serum cholesterol. However, subdividing HDL into its components currently has limited clinical utility, especially given clinical trials have shown no reduction in CVD risk when pharmacologically increasing HDL-C.

Until the utility of modifying these various aspects of HDL function are evaluated clinically, the focus should remain on assessing primary and secondary cardiovascular risk (Central Figure). HDL-C has a marginal role in secondary prevention and the emphasis should remain on LDL-C reduction based on the patient's risk [45]. Low HDL-C should be evaluated with the 10-year ASCVD pooled cohort equation and with management based on current lipid guidelines [46,2]. If HDL-C is elevated, then risk enhancing factors such as apo-B, lipoprotein(a) and high sensitivity c-reactive protein (CRP) levels can be used to refine risk and guide the allocation of lipid lowering therapies. Individuals with diabetes require at least moderate, if not high intensity statin therapy particularly in the setting of high HDL-C. Female specific risk enhancers, such as polycystic ovarian syndrome, gestational hypertensive disorders, and menopause, which have been found to increase the risk of CVD and are associated with dysfunction HDL, should be utilized to guide therapy [23,32,47]. If risk enhancers are present, statin therapy should be considered and imaging based testing should be obtained if the patient has angina. If the patient is without risk enhancers, then lifestyle management alone may be sufficient, as it remains the cornerstone of CVD prevention. Regardless of the level of HDL-C an emphasis on the AHA's “Life's Essential 8″ to reduce CVD risk is paramount: adequate sleep, a healthy diet, exercise, avoid tobacco use and vaping, blood pressure control, lipid and glucose management and a healthy weight [48].

 

## Disclosures and authorship

Dr. Pam Taub reports a relationship with Novartis Pharmaceuticals Corporation, Esperion Therapeutics Inc, Amgen Inc, Novo Nordisk Inc, Sanofi, Medtronic Inc, Edwards, Merck & Co Inc, and Boehringer Ingelheim Pharmaceuticals Inc that includes consulting or advisory.

Dr. Charles German and Dr. Lily Dastmalchi do not have any financial disclosures.

LND conceived of the manuscript, wrote the manuscript, and edited the manuscript. CG and PT appraised and provided critical review of the manuscript.

## Declaration of Competing Interest

The authors declare the following financial interests/personal relationships which may be considered as potential competing interests:

Pam Taub reports a relationship with Novartis Pharmaceuticals Corporation that includes: consulting or advisory. Pam Taub reports a relationship with Esperion Therapeutics Inc that includes: consulting or advisory. Pam Taub reports a relationship with Amgen Inc that includes: consulting or advisory. Pam Taub reports a relationship with Novo Nordisk Inc that includes: consulting or advisory. Pam Taub reports a relationship with Sanofi that includes: consulting or advisory. Pam Taub reports a relationship with Medtronic Inc that includes: consulting or advisory. Pam Taub reports a relationship with Edwards that includes: consulting or advisory. Pam Taub reports a relationship with Merck & Co Inc that includes: consulting or advisory. Pam Taub reports a relationship with Boehringer Ingelheim Pharmaceuticals Inc that includes: consulting or advisory.
